# Amyloidogenesis Abolished by Proline Substitutions but Enhanced by Lipid Binding

**DOI:** 10.1371/journal.pcbi.1000357

**Published:** 2009-04-10

**Authors:** Ping Jiang, Weixin Xu, Yuguang Mu

**Affiliations:** School of Biological Sciences, Nanyang Technological University, Singapore, Singapore; National Cancer Institute, United States of America and Tel Aviv University, Israel

## Abstract

The influence of lipid molecules on the aggregation of a highly amyloidogenic segment of human islet amyloid polypeptide, hIAPP20–29, and the corresponding sequence from rat has been studied by all-atom replica exchange molecular dynamics (REMD) simulations with explicit solvent model. hIAPP20–29 fragments aggregate into partially ordered β-sheet oligomers and then undergo large conformational reorganization and convert into parallel/antiparallel β-sheet oligomers in mixed in-register and out-of-register patterns. The hydrophobic interaction between lipid tails and residues at positions 23–25 is found to stabilize the ordered β-sheet structure, indicating a catalysis role of lipid molecules in hIAPP20–29 self-assembly. The rat IAPP variants with three proline residues maintain unstructured micelle-like oligomers, which is consistent with non-amyloidogenic behavior observed in experimental studies. Our study provides the atomic resolution descriptions of the catalytic function of lipid molecules on the aggregation of IAPP peptides.

## Author Summary

People diagnosed with diabetes have increased from 30 million to 246 million over the last two decades. One hallmark of type 2 diabetes is the formation of amyloid in the pancreatic islet, which is composed of human islet amyloid polypeptide (90%) and lipid molecules (10%). In the long-lasting endeavors against the disease, it is important to understand, at the atomic level, the interaction between peptide aggregation and lipid molecules. In this study, we use molecular dynamics simulations to explore the influence of lipid molecules on the self-assembly process of toxic peptide segments. Moreover, a negative control simulation, employing the non-amyloidogenic rodent sequence, is also performed to evaluate the robustness of the simulation protocol. Our study provides a generic picture of the catalytic role of lipid molecules in the process of amyloidogenesis.

## Introduction

A range of human diseases including Alzheimer's disease, Parkinson's disease, the spongiform encephalopathy and type 2 diabetes mellitus (T2DM) is associated with amyloid deposits of normally soluble proteins or peptides [1]–[3]. In T2DM, the main protein component of fibrillar protein deposits in the pancreatic islets of langerhans has been identified as a 37-residue hormone referred to as islet amyloid polypeptide (IAPP) or amylin [4], which is synthesized in β-cells of the pancreas and cosecreted with insulin [5],[6]. There are convincing evidences that the toxicity of amyloid related diseases may be caused by the soluble intermediate oligomers instead of mature fibrils [7]–[9], and the interaction between lipid bilayer and these soluble oligomer [10]–[14]. For example, channel-like annular structures of oligomers of several amyloidogenic peptides have been observed on the lipid membrane [15],[16], and have been studied by molecular dynamics simulations as well [17],[18]. Moreover, up to 10% components in amyloid deposits from patient tissues were lipid molecules, indicating that the lipids can be uptaken from membranes and then wrapped into fibrillar amyloid [19]–[22]. Most studies so far treated the lipid bilayer as a template to exert its influences on the conformation and aggregation properties of peptides [23]–[26]. There is, however, missing information about how individual lipid molecule involving in the peptide aggregation process. It will then be beneficial to understand the molecular details of how single lipid molecule influences the assembly process of amyloidogenic peptides which is the main focus of the current study.

Besides the external factors, such as lipid bilayer, pH value, the sequences of peptide themselves have great effects on the aggregation behaviors. Several other species such as non-human primates [27], cats [28], raccoons [28], and rodent species (rat [29], mouse [30], hamster [31], etc.) can produce IAPP, but the primary sequence of IAPP varies slightly among species. Importantly, IAPP from rodent species, such as rat/mouse IAPP (rIAPP) lose capacities of aggregating into amyloid fibrils [31], but transgenic mouse models that express human IAPP (hIAPP) develop islet deposits [32]. The rIAPP differs from hIAPP in six amino acids and five of them are clustered in a short decapeptide (residues 20–29), which is considered to be strongly amyloidogenic and forms similar unbranched fibrils itself to the full-length hIAPP [33],[34]. The three proline substitutions in rIAPP20–29 are believed to be highly responsible for the lacking of the amyloidogenic property of the segment or full-length peptide [34]. Although rIAPP has been intensively applied in experimental research acting as a potential peptide inhibitor for peptide aggregation [35],[36], the molecular mechanism of its resistance to amyloid is still not crystal clear. Here, the aggregation of rIAPP20–29 segments is subjected to the same simulation condition as hIAPP20–29 to explore the non-amyloidogenic properties of the peptide and meanwhile to evaluate the simulation results as a negative control.

Due to the metastable and short-lived nature of soluble pre-fibril oligomers at the early steps of fibril formation, experimental data are usually difficult to obtain [37],[38]. Thus, the computational approaches have been employed to complement experimental investigations to gain the insight into the aggregation mechanisms [39]–[44]. Considering multiple copies of peptides needed due to the self-assembly nature of amyloid formation, various simplified representations of molecular systems using implicit solvent models were preferred rather than all-atom models. Santini et al. performed ART-OPEP simulations on trimer of Aβ16–22 by treating side chains as a bead and solvent implicitly [39]. A novel mechanism for single β-strand to surmount unnatural registry without dissociation, referred to as “reptation” was proposed before experimental characterization [45]. Cheon et al. used ProFASi package to reduce the bonded potential energy to include torsional angles only and treated hydrogen bonds explicitly [46]. They were able to carry out two series of 100 Monte Carlo simulations on 20 copies of two fragments Aβ16–22 and Aβ25–35. They observed early-stage events and obtained an atomic-detailed description of “nucleated conformational conversion” (NCC) [47] model for amyloid aggregation. In these studies, simulations were usually started with randomly oriented, extended or random-coiled peptides which underwent *ab initio* folding to form β-sheet oligomers. Albeit simplified models allow studying large-scale systems [46] or observing more events in limited simulation time [39], all-atom explicit solvent models can reproduce amyloid aggregation in aqueous environment more accurately and supply more information on sidechain contacts [48]. Nguyen et al. prolonged a series of conventional MD simulations to 300 ns on Aβ16–22 of 3–6 oligomer size with explicit solvent [49]. The extensive simulations were able to probe the interpeptide sidechain contacts and large conformational fluctuations upon monomer addition to preformed β-sheet oligomers in a “dock-lock” mechanism.

In our studies, an enhanced-sampling method, replica exchange molecular dynamics (REMD) [50] was implemented [51], and all water and peptide atoms are treated explicitly by applying OPLS-AA force field [52]. The four copies of amyloidogenic segment hIAPP20–29 and an extra dioleoylphosphatidylcholine (DOPC) lipid molecule were initially set in extended conformation and dispersed in simulation boxes. The formation of β-sheet containing tetramers, was observed within 100 ns *ab initio* REMD folding simulations. The acquirement of abundant intermediate states suggested two possible β-sheet transition pathways. Simulation of four hIAPP peptides without lipid molecule was also performed. Nonamyloidogenic rat IAPP segments were studied as a negative control with the aim of understanding the inhibitory effect of three proline substitutions.

## Results

### rIAPP20–29 Aggregates Are Amorphous Rather Than Ordered β-Sheet

**Figure 1:**
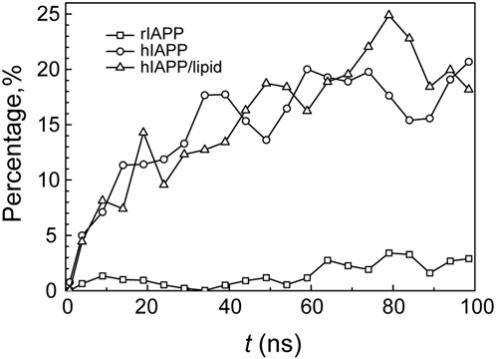
Time evolution of percentages of residues in β-sheet conformation. Residue percentages from all three systems are shown for the lowest-temperature trajectories. The data is averaged every 5 ns.

**Figure 2:**
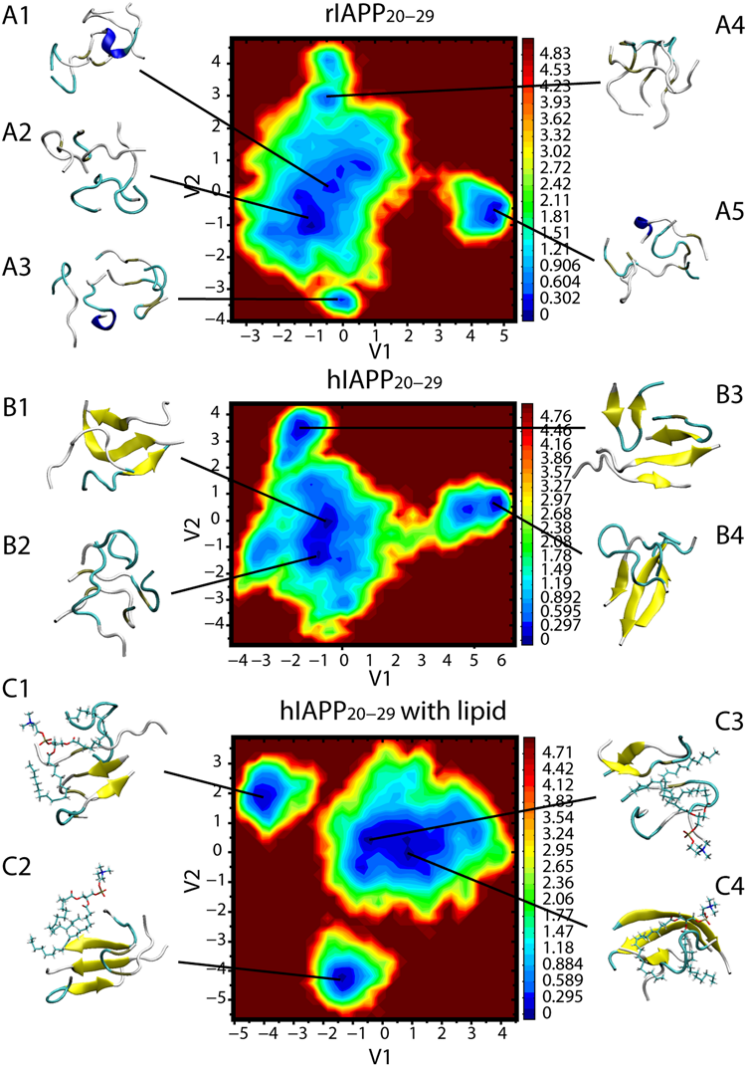
Free energy landscapes (in KJ/mol) of rIAPP, hIAPP, and hIAPP/lipid systems. The energy landscapes are constructed by dPCA method based on the last 80 ns ensemble from trajectories 0, 1, 2, and 3 (four trajectories under lowest temperatures) consisting of 320,000 structures in individual REMD simulations. The representative structures corresponding to each local minimum are plotted by VMD package [54]. Peptides are colored by different sorts of structures, including β-sheet (yellow), helix (blue), turn (cyan), and coil (white). Lipid molecules are also illustrated in C1–C4.

A large amount of experiments have well demonstrated that full-length (37-aa) rIAPP and segment rIAPP20–29 do not form amyloid fibrils *in vivo* or *in vitro* [31],[34],[53]. Time evolution of percentage of residues that adopt β-sheet conformation is shown in Figure 1. Consistent with experimental studies, rIAPP_20–29_ segments seldom exhibit β-sheet structures. Less than 5% residues in the disordered strands adopt β-sheet conformation. Meanwhile, more than 25% residues in hIAPP and hIAPP/lipid participate in β-sheet regions at the end of simulations. On free energy landscapes, the dominant minima are separated by small free energy barriers (Figure 2). The representative structures related to each local minimum are characterized by conformational features according to VMD coloring schemes [54]. Coils and turns are the predominant structure motifs for rIAPP oligomers with a small portion of helices. The whole aggregate is compact and single strand twists to form a coil without long extended β-structure portion. In contrast, quite ordered β-sheet dimers or trimers are the representative of hIAPP snapshots. In the eight hIAPP representative snapshots (B1–B4 and C1–C4), both parallel and antiparallel H-bonding patterns are observed. And the mixture of ordered and amorphous structures in the hIAPP ensemble illustrates the dynamical equilibrium between the two states. The ensemble statistics from dPCA results and time series of β-sheet percentages both suggest the fact that after 100 ns simulation, while rIAPP_20–29_ aggregates remain disordered, hIAPP oligomers are on divergent ways to form amyloid nuclei in the form of β-sheet dimer, trimer, or even tetramer.

Of interest is that the decrease of Cα-atom radius of gyration (Rg) is much faster than the increase of length of β-sheet regions in four hIAPP20–29 strands. Within 5 ns, hIAPP20–29 Cα-Rg rapidly drops from initially ∼1.6 nm to ∼1.1 nm and continues to slowly decrease to ∼0.95 nm in the following 90 ns. Nevertheless, only 5% hIAPP residues are transformed into β-sheet structure and β-sheet composition reaches a relatively stable level (∼20%) after 50 ns. These early stage (5–50 ns) intermediate species are condensed (small Rg) but less structured (low percentage of β-sheet regions), which may be the amorphous aggregates described in other's simulations [46],[55],[56] as well as experiments [45],[47]. The rapid collapse of initially dispersed strands is followed by a slow structural reorganization to allow amorphous species to transform into ß-sheet oligomers which can act as potential nuclei on the way to higher-level aggregates.

### Antiparallel and Out-of-Register Patterns of β-sheet Oligomers

**Figure 3:**
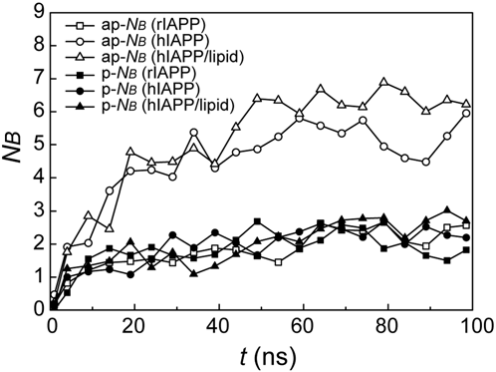
Time evolution of average number of antiparallel (ap) and parallel (p) β-bridges (N_B_). The numbers are averaged every 5 ns from the lowest-temperature trajectories.

**Figure 4:**
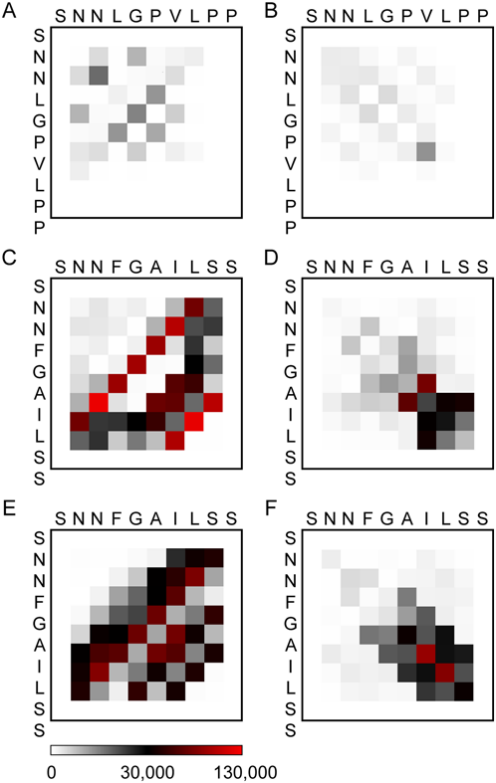
β-bridge occurrence contact map. (A) and (B), (C) and (D), and (E) and (F) count rIAPP, hIAPP and hIAPP/lipid, respectively. Left and right panels count only antiparallel and parallel β-bridges, respectively. The contact map is colored by the occurrences of residue pairs which make inter-strand β-bridge. Simulation data from the last 80 ns are used.

Several experimental studies have found that unlike full-length hIAPP, amyloid fibrils constituted by fragment hIAPP_20–29_ contain both antiparallel and parallel β-sheet structure by using FTIR (Fourier transform infrared spectroscopic) [57] and ssNMR (solid-state NMR) [58]–[60] techniques. Although both parallel and antiparallel β-sheets are observed in representative snapshots, the two opposite orientation patterns are found to have different occurrences by monitoring number of antiparallel (ap-N_B_) and parallel β-bridges (p-N_B_) during simulation course. From Figure 3, ap-N_B_ and p-N_B_ increase at different rates and eventually ap-N_B_ is more than two times favored than the parallel pattern. A β-bridge occurrence contact map which is constructed to disclose the detailed information of β-strand alignment patterns indicates the same orientation preference of the decapeptide (Figure 4). In principle, numbers of counts from left panels (antiparallel β-bridges) are overwhelmingly more than those from right panels (parallel β-bridges) indicating antiparallel β-sheets are much preferred over parallel sheets. Such observation was also found in a recent Monte Carlo simulation: when the aggregation size is small, the fraction of antiparallel β-sheets is dominant [48].

Furthermore, the registry patterns of interacting strands within one β-sheet layer are demonstrated clearly in contact maps. Both parallel and antiparallel β-sheets exhibit a mixture of in-register and various out-of-register patterns. Although the tetrameric oligomers are partially ordered in β-sheet conformation, no uniform alignment patterns are found to be more favorable than others. The in-register patterns are able to extend β-sheet to a longer length than that of out-of-register patterns. The out-of-register patterns are more often found in antiparallel orientation than in parallel pattern. For hIAPP and hIAPP/lipid regardless of parallel and antiparallel patterns, the C-terminal region contributes more in the β-bridge formation. It is not surprising to find that rIAPP has much less β-bridge contact counts considering its nonamyloidogenesis nature.

### Critical Residues to β-sheet Formation or Disruption

**Figure 5:**
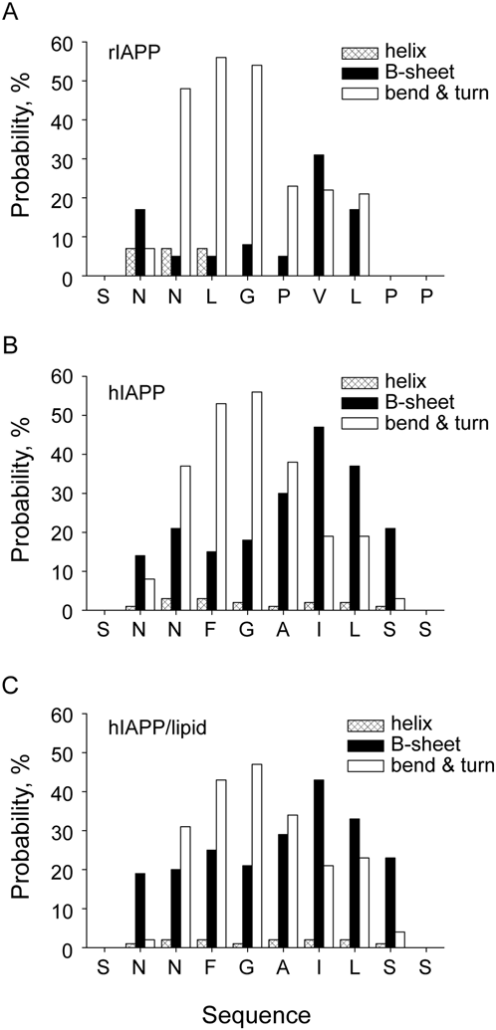
Secondary structure propensity of residues in rIAPP_20–29_ and hIAPP_20–29_. The secondary structures are assessed by DSSP package.

To investigate the roles that residues play in aggregation, secondary structure propensity (SSP) for the ten residues are analyzed (Figure 5). It is obvious that three hydrophobic residues A25, I26, and L27 in hIAPP_20–29_ show high propensity for β-structures. The hydrophobic region (residues 25–27) is considered to be the core part of β-sheets for fragment hIAPP_20–29_ by experiments [57],[58]. The terminal residues (S20, N21, and S29) are generally unstructured, as their preferences for any of the three sorts of secondary structures are very low. Residues N22, F23, and G24 show high propensity for turn/bend. This may be due to the higher backbone flexibility of G24, and the side chain of F23 can be helpful for stabilizing turn/bend structures. The whole hIAPP_20–29_ sequence shows a rather low propensity for helical structures. In the presence of lipid (Figure 5 C), the probabilities of residues 22–24 taking turn/bend structures are reduced by approximately 10%, and their probabilities for β-sheets are increased contrastively. Moreover, fewer occurrences of β-hairpin strands are found in the presence of lipid molecule. Consequently, a role of lipid molecule in the aggregation process is disclosed that it prevents peptide from the formation of monomeric hairpin structure and helps the peptide stay in extended conformation. Compared to hIAPP, rIAPP fragment shows a similar propensity for turn/bend in residues 22–24, but β-structure possibilities of the whole sequence greatly decrease with only those of V27 and L28 remaining a relatively high level. Single mutation I27V slightly reduces ability of hIAPP_20–29_ [34] to form amyloid fibrils probably because that a valine residue has a nearly same hydrophobicity and SSP as an isoleucine residue does.

### Disrupting Roles of Proline Residues

**Figure 6:**
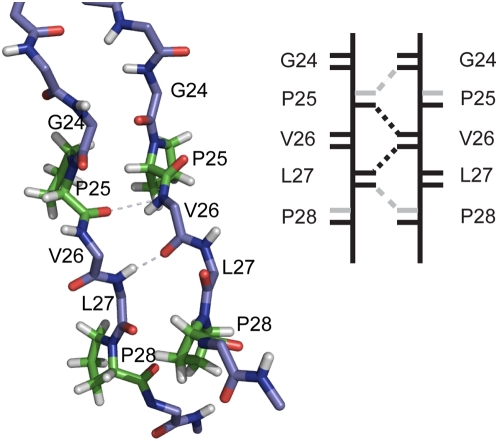
Backbone hydrogen bond interaction on C-terminal region of two strands of rIAPP. Only mainchains of residues (except four prolines) are shown in stick by PyMOL package. Four prolines (two P25, and two P28) are shown in green color. Hydrogen bonds between Val26 and two interstrand residues Pro25, Leu27 are presented as dash lines. The right illustrative figure gives a simple demonstration of hydrogen bonding network of the structure. Short black lines indicate hydrogen bond donors (amino groups) and acceptors (hydroxyl groups); short grey lines indicate the missing hydrogen bond donors on prolines. The real hydrogen bonds in structure are shown in black dash lines on the right; the grey dash lines show the missing hydrogen bonds which are supposed to form between prolines and their partner residues.

Figure 6 presents a snapshot of the region on the C-terminals of two rIAPP strands from the simulation. The two rIAPP strands are in a perfect in-register alignment with only one parallel β-bridge between two Val26 amino acids. This alignment pattern makes a large contribution to rIAPP sheet alignments (Figure 4B). The snapshot offers hints about influence of two prolines (P25, P28) on the interstrand hydrogen bonding network. As illustrated in the sketch plot, V26 residues form stable interstrand H-bonds but the extended H-bond ladder is disrupted by the missing hydrogen atoms on proline amide groups. The occurrence of β-bridges between V26 reaches a large number of over 10000 counts compared to around 2000 counts of other residue pairs in Figure 4. The other pairs (G24, P25, and L27) on the same alignment pattern show a zero β-bridge count. Similarly, the contact numbers around prolines in either parallel or antiparallel patterns are at a comparatively low level, indicating that prolines fail to form H-bonds in nearly all alignment patterns. Both the β-bridge contact maps of rIAPP and the parallel, in-register dimer snapshot describe the same story that the failure of proline to be H-bond donor prevents extension of β-bridges and therefore avoids formation of stable β-sheets.

In addition to the disruption of continuity of H-bonds caused by proline, the uniform backbone structure within β-sheets is also perturbed. In Figure 6, amide and carbonyl groups of the G24 which sits before P25 lose their appropriate positions for H-bond formation. The cyclic structure of proline side chain limits its ϕ backbone dihedral angles at a small range between −90° to −60°, which brings an extra conformational rigidity to its structure and makes proline a structural disruptor in secondary structure elements such as α-helices and β-sheets. We find that proline dihedral angle ϕ is restrained to a narrow range which cannot accommodate β-sheet structure (Figure S2). The distributions of ϕ dihedral angle of the three prolines (P25, 28, 29) on rIAPP as well as their counterpart residues on hIAPP and hIAPP/lipid show a clear difference. As a rule, the ϕ angle for a β-sheet structure is about −120° to −140°. The counterpart residues on hIAPP all have a considerable probability for ϕ angle in the range between −120° to −140°. However, ϕ angles of three prolines locate in an extremely narrow range (−90° to −60°) with little overlapping region with β-sheet structure. Thus backbone structure around prolines would induce considerably unfavorable high energy if it adopted a β-sheet conformation.

To examine how the disordered rIAPP aggregates lacking of backbone H-bonds can be stabilized, an ensemble of 100 structure snapshots has been extracted from the region with the lowest free energy (free energy = 0 on the free energy landscape) for calculating binding energy. The binding energy was calculated by MM/GBSA method and is specified in method section. The binding energy for hIAPP was also estimated for comparison. The breakdown of binding energy components is listed in Table 1. It is shown that the amorphous rIAPP oligomer configuration can be stabilized at a comparable level to hIAPP (with similar ΔE_total_). For both IAPP segments, the inter-peptide interaction (with negative ΔE_vdw_ and ΔE_elec_) contributes the oligomerization favorably, while the polar solvation energy is unfavorable (positive ΔE_gb_). The difference of ΔE_elec_ between rIAPP and hIAPP, 122.5 kJ/mol, and the difference of ΔE_gb_, −96.5 kJ/mol correlates with the fact that there is less backbone H-bonding interaction within rIAPP oligomer, and relatively favorable solvation energy for rIAPP. Overall, aggregation of both peptides is driven by nonpolar interaction.

**Table 1:** Decomposition of the average binding energy of the highest populated tetrameric oligomers for rIAPP and hIAPP.

*Binding Energy (kJ/mol)*	*hIAPP*	*rIAPP*
ΔE_vdw_	−462.2±56.60	−493.3±40.49
ΔE_elec_	−377.1±86.51	−254.6±52.73
ΔE_gb_	471.7±135.41	375.2±87.09
ΔE_surf_	−38.0±5.03	−36.8±2.83
ΔE_p_	94.6±99	120.6±90
ΔE_np_	−500.2±61	−530.0±43
ΔE_total_	−405.6±100	−409.4±98

E_vdw_, and E_elec_ are the van der Waals and electrostatic binding terms. E_gb_ and E_surf_ are the solvation energies of polar and nonpolar residues, calculated by Amber 9 using the Generalized Born model. E_p_ and E_np_ are the sums of polar energy (E_elec_+E_gb_) and nonpolar energy components (E_vdw_+E_surf_), respectively. E_total_ is the sum of E_p_ and E_np_. Error bars represent standard deviations over 100 configurations extracted from the global minimum indicated by the free energy landscapes.

### Conformational Reorganization of Pre-nucleus Oligomers in Two Pathways

The nucleation process of four hIAPP_20–29_ strands in this study involves complex structural transition from initial amorphous oligomer states to highly ordered β-sheets. The fundamental element of structural transition is the backbone hydrogen bond formation. Thus the number of extended β-bridges (N_B_) should be a suitable reaction coordinate for describing the conversion process. The ordered oligomer state, namely β-sheet dimer, trimer and tetramer, can also be used to describe the degree of order of an ensemble of structures. The evolution of different β-sheet oligomerization state as a function of N_B_ is elaborated based on the ensemble trajectories at low temperatures. Besides, following a replica trajectory that contains information of a continuous structural evolution, the transition between an amorphous state and an ordered state can be vividly demonstrated.

**Figure 7:**
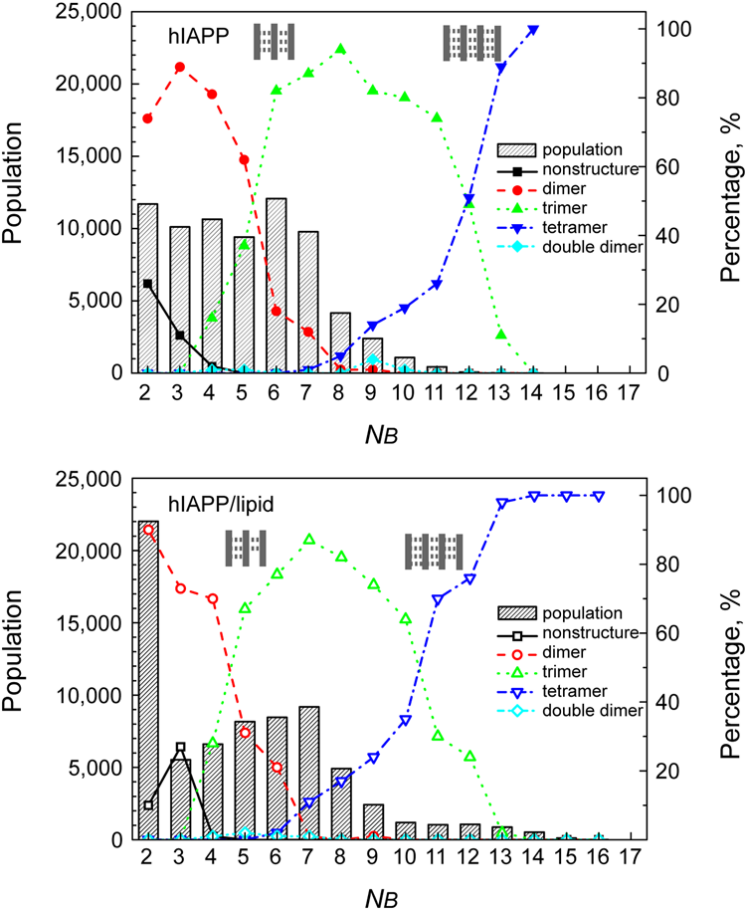
Population (in bars) and percentage of different oligomer sizes (in lines and symbols) of ensembles with fixed value of N_B_.

The number of N_B_ is depicted by bar chart in Figure 7 for both hIAPP and hIAPP/lipid systems. The percentages of different β-sheet oligomer states in an ensemble with a fixed N_B_ are plotted as symbols. The population of ensembles with N_B_ value in the range of 2 to7 is large. Those ensembles with large N_B_ (more than 7) are highly ordered but have a relatively small population. The dominant β-sheet oligomer size changes gradually with the increase of N_B_: unstructured→dimer→trimer→tetramer. The increase of β-sheet oligomer sizes also indicates the transformation from amorphous aggregates to a more ordered state. Such transformation is realized by monomer addition. The ensembles which have two separate dimers are found to be hardly populated.

It is surprising to discover that even when N_B_ is small, the percentage of trimer is larger than that of dimer (e.g. N_B_ = 6 for hIAPP, and N_B_ = 5 for hIAPP/lipid). Similarly, the percentage of tetramer is larger than that of trimer when N_B_ = 12 for hIAPP, and N_B_ = 11 for hIAPP/lipid. For a clear description, at the transition point, four illustrative sketches for trimeric and tetrameric sheets are drawn, with short dashed lines denoting single β-bridges. Only three out of the ten residues in each strand forming hydrogen bonds are competent to stabilize a tetrameric sheet. This indicates that the β-sheet nucleation site is not necessary to have a long and perfect in-register pattern; a short β-sheet region is capable of being a template to invite free monomers to join the nucleus. The “template” hypothesis was inspired and supported by the work of Kameda and Takada [61], as the hydrogen bond donors and acceptors on the template are in perfect positions for hydrogen bond forming with another monomer. An interesting difference between two hIAPP systems is that for hIAPP/lipid, it always needs less value of N_B_ to develop structures with higher degree of order. And hIAPP/lipid system has more structures with large N_B_. Clearly the presence of lipid molecule helps to stabilize the ordered structures and therefore accelerates the emergence of higher order of β-sheet oligomer.

**Figure 8:**
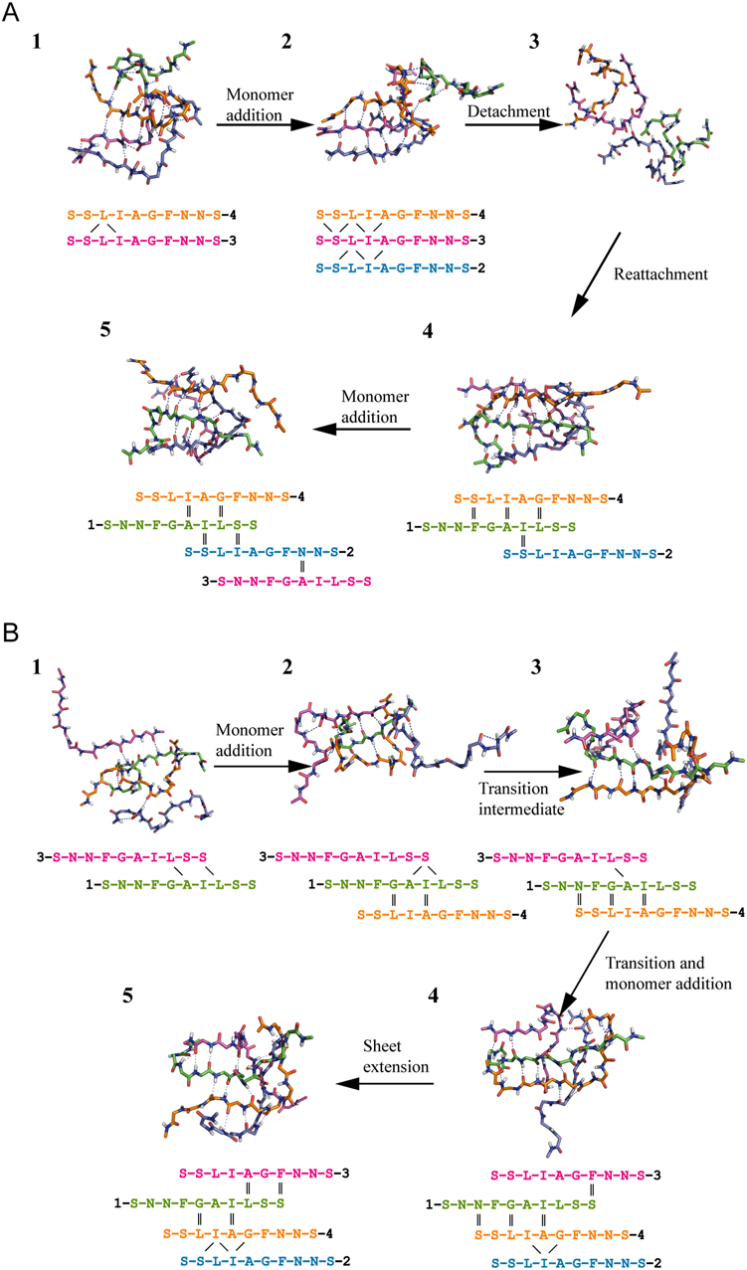
Two transition pathways illustrated by replica trajectories of hIAPP. (A). replica 2; (B). replica 20. Only main chain atoms of peptides are shown in sticks. Dashed blue lines indicate hydrogen bonds. Four strands are distinguished by different colors (strand 1, 2 3 and 4 is in green, blue, magenta and orange color, respectively). The alignment patterns are also sketched beneath corresponding snapshots. The strand numbers are labeled at the N-terminals. Hydrogen bonds involved in the predominant β-sheet regions are marked, with antiparallel hydrogen bonds labeled in double short lines, parallel hydrogen bonds labeled in forward/back slashes.

We have examined several folded replica trajectories, and key intermediate states from two hIAPP trajectories are shown to demonstrate the detailed transitions from amorphous oligomer to ordered β-sheet oligomer in Figures 8. Among all folded trajectories we have traced, some of the aggregation pathways are simple and straightforward, in which the increase of the β-sheet oligomer size is simply through monomer addition and sheet extension accomplished by forming more hydrogen bonds between the two β-strands. Other trajectories show much more complicated pathways and involve more reorganization process such as detachment/reattachment of the aggregates (Figure 8A) and conformational reorganization such as parallel to antiparallel transition (Figure 8B). In Figure 8A, snapshot 2 is a β-sheet trimer with parallel in-register H-Bond pattern. It undergoes a complete detachment process. All the H-bonds are lost in snapshot 3. The reattachment finishes in snapshot 4 where a new β-sheet trimer is formed. The three strands involved (1, 2 and 4) are different from the previous ones (2, 3 and 4). The H-bond pattern is changed to antiparallel. When the structure evolves to snapshot 5, a new strand is added to the trimer and a tetramer is formed. The transition from parallel sheet to antiparallel sheet has also been captured in replica trajectory B. Such transition does not need a complete detachment process as in trajectory A. It involves only internal reorganization as trajectory B shows. In this case, one single hydrogen bond in the parallel pattern remains and the whole strand rotates by 180° around the hydrogen bond. Afterwards the newly generated antiparallel hydrogen bonds will form near the place of the original hydrogen bond.

### Lipid Stabilizes β-sheet Oligomers by Binding to a Hydrophobic Cluster

**Figure 9:**
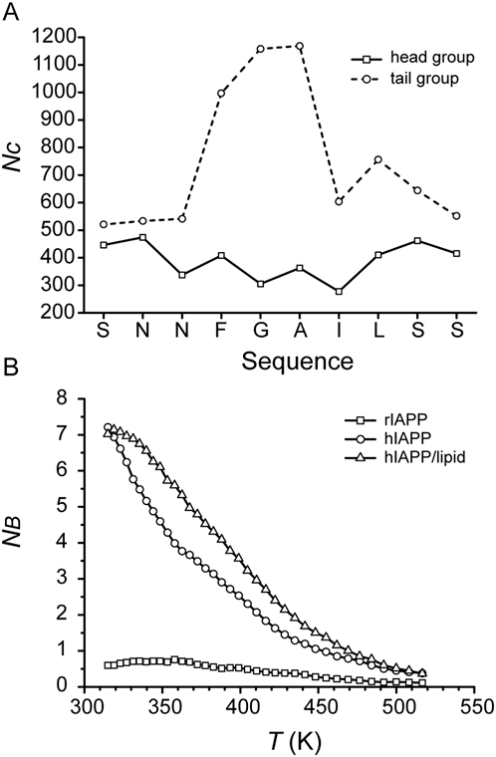
The influence of lipid on peptide aggregation. (A). Occurrences of atomic contacts (N_C_) between heavy atoms on lipid head (square symbol, sold line) or tail (circle symbol, dashed line) groups and Cα atoms on hIAPP fragment. Distance cutoff is 0.6 nm. The counts are scaled by the total atom number of head or tail group in order to prevent the bias favored by large atom number of tail group. (B). Temperature dependence of number of β-bridges N_B_ averaged over the ensemble trajectories at the same temperature.

The lipid-associated peptide toxicity and aggregation enhancement has been widely established under a variety of lipid models such as micellar [62] and bilayer membranes [25], even free fatty acids and lipids [63],[64]. The mature amyloid fibrils are found to contain a portion of lipids which are supposed to be taken up from membranes and wrapped together with peptide while aggregation goes on [25]. Based on results discussed previously, the presence of a lipid molecule has clear effects on the aggregation process of hIAPP peptides: The propensities for β-sheet structure of residues 20–23 in hIAPP/lipid system are increased and their propensities for turns and bends are correspondingly reduced (Figure 5); the value of N_B_ needed for the formation of β-sheet oligomers is consistently reduced by one in the presence of the lipid molecule (Figure 7). To probe the lipid-peptide binding manners in a statistical way, the occurrences of atomic contacts (N_C_) between heavy atoms on lipid head/tail groups and Cα atoms on hIAPP fragment are calculated (Figure 9A). All residues have similar probabilities of contacting with head group. The general pattern of such contact is the H-bonds formed between head group and polar side chains. In contrast, the probabilities of contacting the tail group for different residues are quite distinct. Nonpolar residues show obviously higher inclination, especially for residues F23, G24, and A25, indicating a specific lipid-binding site. The binding site is exactly the region which has high SSP for turns and bends in the absence of lipid (Figure 5). The increased propensity for β-sheet of this region in hIAPP/lipid (Figure 5C) is due to the specific binding of nonpolar lipid tails.

**Figure 10:**
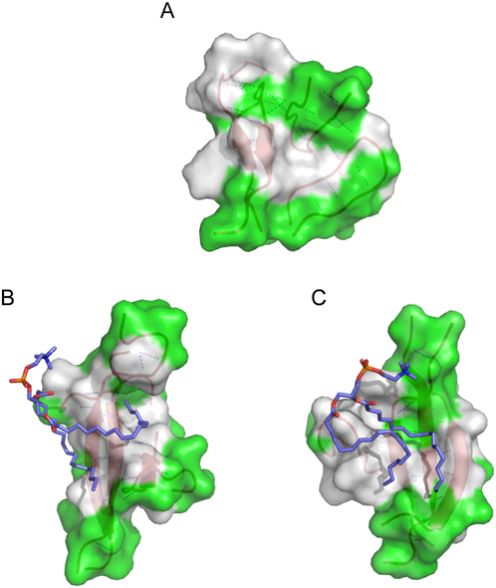
Structural models of lipid- peptide oligomer interaction. (A) One snapshot from hIAPP having 14 β-bridges, (B) and (C) two snapshots from hIAPP/lipid with 16 β-bridges. Peptides are shown in cartoon, red and dashed lines indicate the backbone hydrogen bonds. White color indicates hydrophobic surface (residues F23, A25, I26, L27), green color indicates polar surface. In structures (B) and (C), tails of lipid molecule (in stick, blue) bind to the hydrophobic surfaces.

The binding of lipid molecule functions in another critical way that helps to stabilize the ordered conformation of β-sheet oligomers. Figure 9B describes temperature dependence of average number of β-bridges, N_B_. N_B_ decreases slowly to zero when the temperature increases to over 500 K. In nearly all temperature range the average N_B_ of hIAPP/lipid is more than that of hIAPP. The melting temperature (where N_B_ = 3.5) is 380 K without lipid and increases to 400 K in the presence of lipid. It is well known that amyloid β-sheet structure is stabilized not only by backbone hydrogen bonds network and also by close side chain packing [65],[66]. In Figure 10, three structures with the largest number of extended β-bridges from two hIAPP simulations are shown. Nonpolar surfaces are coalescent into a patch because the hydrophobic residues are prone to pack with each other (Figure 10 A, in the absence of lipid molecule). With the presence of lipid molecule, the lipid molecule is selectively docked onto the hydrophobic patches. The β-sheet region is undoubtedly stabilized through lipid binding on such hydrophobic patch (Figure 10 B, C) which makes the hydrophobic clusters dissociate much more difficult. This also explains why less value of N_B_ is needed to maintain β-sheet oligomer in hIAPP/lipid system (Figure 7).

## Discussion

The difference of five residues between hIAPP and rIAPP in the core region exerts evident effects on aggregation characteristics, among which three proline substitutions have the strongest influences. Proline is commonly found in turns exposed to solvent, which may benefit from its rigidity that costs less entropy penalty upon folding. The cyclic structure of side chain makes proline not compatible to any secondary structures, but it is occasionally found as the first residue of α helices and in the edge strands of β sheets to prevent protein self-assembly. In an atomic detailed level, we have studied how the special structure features of proline, including lacking of amide hydrogen atom and ϕ dihedral angle which is not overlapping with β-structure, influence the aggregation ability of IAPP20–29. The missing hydrogen atoms on proline backbones disrupt the H-bonding network and therefore the β-sheet stability is weakened. Besides, the rigid backbone of proline induces unfavorably high energy to β-conformation. The two reasons explain the loss of amyloid aggregation ability of rIAPP_20–29_ brought by proline mutation. Comparatively, effects of the other two residue mutation (F23L and I26V) are indifferent. Their SSP greatly resemble that of counterparts on hIAPP and a certain amount of backbone H-bonds are formed among the two variant residues, indicating a less important function in abolishing rIAPP20–29 aggregation.

Recent NMR measurements have provided several constraints on hIAPP protofilaments with striated ribbon morphologies [65]. The basic structural unit of the model contains two layers in a C2 rotational symmetry about fibril axis, and the peptide forms parallel H-bonds to adjacent β-strand within each sheet. Unlike 37-aa hIAPP, fragment 20–29 shows obvious antiparallel H-bonding preference without a clear and uniform strand alignment configuration which may arise from structural heterogeneity or polymorphism in amyloid fibrils [57]–[60]. A most recent ssNMR[60] study suggested an antiparallel pattern with the central FGAI region in registration (F23 H-bonded to I26). We also observed the same alignment pattern mixed with in-register and other out-of-register patterns. It is reasonable that no uniform or dominant alignment pattern was observed if merely peptide tetramer was studied in 100 ns simulation, as the oligomer size is inadequate to form a stable nucleus for β-sheet elongation. It can be predicted that, similar to the ways of parallel-to-antiparallel transitions that occurred in our simulation, the pre-nucleus tetramer will undergo conformational reorganization and adopt a uniform alignment pattern so long as both the number of oligomers and simulation time exceed a critical values.

The fragment 20–29 was thought to form a highly ordered hydrophobic core in fibrils. Nevertheless, recent studies by ssNMR [65] and X-ray [67] indicate an obvious bend around residue G24 in mature fibrils derived from full-length hIAPP, which most probably arises from small-sized Gly and aromatic ring of Phe23 nearby. A similarly conformational preference of the segment in membrane-mimicking environments was also found by solute state NMR[68] and MD simulation study[69]. We also found that the SSP of F23, G24, and A25 for bend and turn indeed is higher than that for β-sheet structure. Moreover, we have observed significant occurrence of hairpin conformation in a monomeric form. Due to the limited size of the peptide segment and the lacking of other stabilizing factors, the hairpin monomer is likely to be only a transient form in aqueous environment. NMR observation also supports a linear β-strand for fragment hIAPP20–29[60]. We found that the probability of hairpin emergence can be reduced by lipid interaction at a specific binding site at positions 23–25. The lacking side chain on G24 and the large nonpolar side chains of neighboring F23 and A25 comprise a perfect hydrophobic cavity on peptide surface for lipid tail embedding inside. The embedded lipid reduces the backbone flexibility of G24 and renders the segment in linear β-strands, which accounts for the increased propensities for β-structure of only residues 23, 24, and leaving SSP of other residues mainly unchanged in the presence of lipid molecule.

The experimentally observed sigmoidal profile of fibrillogenesis kinetics is normally interpreted by a nucleated growth mechanism [47],[70]. The self-assembly kinetics is characterized by an initial lag phase (nucleation) which is assumed to be the time required for a “nucleus” of critical size to form. This is followed by an exponential growth phase (elongation) where fibril growth proceeds rapidly by association of monomers or oligomers to the nucleus. By probing the aggregation behavior of Sup35, Serio *et al.* has proposed a revised nucleated growth mechanism NCC model which depicts that nuclei form through conformational rearrangements within micelle-like, structurally dynamic oligomers [47]. The condensed but disordered pre-nucleus species were probed in our and others' simulations/experiments [46],[47],[55],[56],[70]. These amorphous oligomers formation are mainly driven by hydrophobic effects. The competition between hydrophobicity and backbone H-bonding is believed to be a major determinant of aggregation process [46]. In our simulations, β-sheet dimers were generated under the help of the hydrophobic residues. As a β-sheet template of minimum size, dimers facilitate isolated monomeric peptide in solution to participate in the nucleus. Majority of the disordered-to-ordered conversions occurs without fully dissociation of the early-stage molten oligomers. Thus the aggregates sustain a low Rg (radius of gyration) throughout the conversion processes. These indicate that the conformational rearrangements from amorphous to nucleus-competent oligomers involve mainly internal reorganization which is consistent with the “reptation” mechanism [39],[45].

Although the appearance of β-sheet dimers can perform as the starting point of peptide aggregation, monomer addition is unfavorable until the nucleus reaches a critical size according to nucleated growth mechanism [71]. This brings a question on how high-energy pre-nucleus β-sheet oligomers can be stabilized in aqueous environment. In our 100 ns simulations, the final tetramers are partially ordered with only 25% residues in β-sheet conformation. The terminal residues, as indicated by SSP, hardly join in the β-sheet core. They interact with intrastrand or interstrand residues through polar contacts on side chains. The formation of backbone H-bonds constitutes less than 50% of overall hydrogen bonds. The hydrophobic side chains tend to cluster into patches in order to minimize the exposed nonpolar surface area. In the presence of a lipid molecule, the hydrophobic tails additionally help to stabilize the unstable short β-sheet dimers and trimers by specific binding to nonpolar patch. In conclusion, pre-nucleus species prefer a partially ordered structures rather than a perfect extended β-sheet conformation. These partially ordered short β-sheet oligomers comes from the process of repeated detachment/reattachment or internal reorganization to search for the most preferred orientation and alignment patterns. This explains why aggregation process can be promoted by free lipids without a membrane or micellar surface for peptide to concentrate on [63],[64].

In summary, the present all-atomic REMD simulations suggest an explanation on how the proline substitutions influence the amyloid aggregation capacity of rIAPP20–29. Preference for antiparallel interstrand orientation and the lack of uniform registration alignment are the two characteristics of early-stage per-nucleus oligomers. The rapid-collapsed amorphous aggregates can evolve to partially ordered β-sheets through conformational rearrangements and two pathways of parallel-antiparallel transitions are traced. Meanwhile, key residues which are responsible for either β strand formation (A25, I26, and L27) or lipid binding (F23, G24, A25) are recognized. The specific interaction between lipid tails and hydrophobic residues is found to stabilize the β-sheet region, indicating a catalysis role of lipid molecule in hIAPP peptide self-assembly. These findings are applicable to other types of amyloidogenic peptides and indicate a general pattern of interaction between lipid and amyloidogenic peptides [72],[73]. Interestingly, a similar specific lipid-hydrophobic residues interaction has also been resolved for explaining the toxicity action of antimicrobial peptides [74],[75].

## Materials and Methods

### Simulation Setup and Protocol

The peptide segments r/hIAPP20–29 and dioleoylphosphatidylcholine (DOPC) molecule were represented by all-atom OPLS-AA force field [76],[77] and solvated by explicit SPC water molecules. Totally three REMD simulations were performed. For abbreviation, rIAPP, hIAPP, and hIAPP/lipid will be used to represent the simulation systems with 4 rIAPP_20–29_, 4 hIAPP_20–29_, and 4 hIAPP_20–29_ together with DOPC lipid molecule, respectively. The peptides capped by ACE and NME groups in N and C terminals were initially constructed in a fully extended conformation and separated by at least 2 nm from each other to avoid interaction bias. The four identical peptides (rIAPP20–29 or hIAPP20–29) in each system were arranged in parallel or mixed parallel/antiparallel patterns to include all four possible arrangements: (i) N-terminals of all four peptides were placed upwards; (ii) N-terminals of three out of four peptides were placed upwards; (iii) N-terminals of two peptides on one side were placed upwards, and (iv) N-terminals of two peptides on the diagonal directions were placed upwards. The four starting structures in different arrangements were alternately used as the initial frames of 36 replicas to avoid bias in favor of parallel or antiparallel β-sheet alignments during REMD simulations. The initial configurations are shown in Support Information, Figure S1. The DOPC lipid molecule in an extended state was aligned along the center axis of the box, parallel to the linear peptides. The peptides in each system were solvated in a 4*4*4 nm cubic box of SPC water, keeping a minimum distance of 1 nm between the solute and each face of the box. The final setup of each system contained 1833 SPC water molecules for rIAPP system, 1841 SPC water molecules for hIAPP system and 1782 water molecules for hIAPP/lipid. All systems were neutral and no extra counterions were added.

The GROMACS program suite [78] and OPLS-AA force field [76],[77] were used in all three systems. The parameters for bonded and non-bonded interactions of DOPC lipid molecules were derived from related OPLS force field. All bonds involving hydrogen atoms were constrained in length according to LINCS protocol [79]. Electrostatic interactions were treated with particle mesh Ewald method [80] with a cutoff of 0.9 nm, and a cutoff of 1.4 nm was used in the calculation of van der waals interactions. The integration time step of simulation was set to 0.002 ps. The protein and the water groups were separately coupled to an external heat bath with a relaxation time of 0.1 ps. Non-bonded pair lists were updated every 5 integration steps (0.01 ps). After 500 steps of steepest-descent minimization, the REMD simulations continued for 100 ns. The temperatures in the REMD simulations were ranged from 315.0 K to 516.7 K, and proper temperature intervals were selected to result in approximately 30% averaged exchange possibility for each replica. Exchanges between neighboring replicas were tried every 1000 steps (2 ps) and the conformation coordinates were output every 500 steps (1 ps). After 100 ns REMD simulation, each system generated an ensemble of 100,000 structures at each temperature and total 3,600,000 structures at all temperatures.

### Secondary Structure Assessment

The DSSP algorithm written by Wolfgang Kabsch and Christian Sander was used to identify secondary structure conformation of β-sheet oligomers [81]. The algorithm is mainly based on identification of H-bonding (hydrogen-bonding) patterns. The identification of H-bonds is relied on calculating electrostatic interaction energy between H-bond accepter C, O and donor N, H atoms. A good H-bond has about −3 kcal/mol interaction energy. Here, a generous cutoff is chosen (if 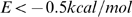) and well tested to allow for an N-O distance up to 2.2Å. Depending on the H-bonding patterns, DSSP recognizes mainly seven types of secondary structures which can be grouped into three classes: helix (α-helix, 3_10_-helix, π-helix), β-strand (isolated β-bridge, extended β-sheet) and loop (turn, bend). β structures are the dominant secondary structures in our aggregation simulation. β-bridge is the basic unit of β-sheet. Either a parallel or antiparallel β-bridge forms between residues i and j, if there are two H bonds between two nonoverlapping stretches of three residues each, i−1, i, i+1 and j−1, j, j+1. Then β-sheet can be defined accordingly as a set of consecutive β-bridges of identical type (parallel or antiparallel). In our study, the size of a β-sheet oligomer is defined more strictly as following: β-sheet dimer is formed only when two β-strands connected by a minimum of two β-bridges (instead of one β-bridge according to DSSP default definition); β-sheet trimer is defined as only one β-strand connected by two other β-strands in the same mode; similarly β-sheet tetramer is identified if two β-strands are connected to two other β-strands respectively.

### Dihedral Angle Principal Component Analysis (dPCA)

A modified PCA version, referred to as dihedral angle PCA or dPCA, was used to represent the conformational distribution on the free energy landscape [82]. In dPCA measurement, only backbone dihedral angles are considered; other internal fluctuations (such as bond lengths, bond angles, etc.) and overall motions are efficiently removed because they contribute comparatively little to the fold of peptide. The method is more appealing than traditional PCA specifically for amyloid aggregation. The reason is that conformational transition into β-sheet during this process can be reflected by variation of backbone dihedral angles, instead of sidechain configuration. After free energy landscapes are plotted, the representative structure of individual local minimum is chosen as following: the structures with their V1 and V2 components close to the local minimum are selected; then a clustering method based on pair-wise RMSD is applied; usually a group with a dominant population emerges; the structure which is the center of the group is assigned to the representative structure. The RMSD cutoff is 0.2 nm for peptide backbone atoms. Here the combination of dPCA and clustering has overcome the limitation of each method: the heterogeneous ensemble in local minima of dPCA is screened by clustering method; the structural ensemble with a large population which cannot afford to be grouped by clustering method is easily analyzed by dPCA.

### Binding Energy Calculation

The binding energy of tetrameric oligomers was estimated by equation:
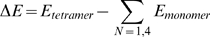


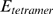
 and 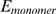 are the energies of tetrameric oligomer and individual monomer, respectively, both are consisting of two terms: one is peptide vacuum potential energy calculated by GROMACS package and the other is solvation energy estimated by using generalized Born (GB) model in the sander module of AMBER 9 [83]. The source code of the tleap program was modified to allow the use of OPLS-AA force field. The modified GB model used was developed by A. Onufriev, D. Bashford and D.A. Case [84].

## Supporting Information

Figure S1 Initial arrangements of 4 identical hIAPP_20–29_ peptides. Backbones of peptides are shown in cartoon; side chains of Phe23 are explicitly represented in sticks for readily identification of N-terminals. (A) N-terminals of all four peptides point upwards; (B) N-terminals of three out of four peptides point upwards; (C) N-terminals of two peptides on one side point upwards; (D) N-terminals of two peptides on the diagonal point upwards.
(0.94 MB TIF)

Figure S2 Distribution of ϕ dihedral angle of three prolines on rIAPP (black curves) and their counterpart residues on hIAPP (red curves) and hIAPP/lipid (green curves).
(4.90 MB TIF)
