# Supplementary figures and images for: Amyloidogenesis Abolished by Proline Substitutions but Enhanced by Lipid Binding
Source: PLoS Comput Biol. 2009 Apr 10;5(4):e1000357. doi: 10.1371/journal.pcbi.1000357 (PMC2663790; doi:10.1371/journal.pcbi.1000357)

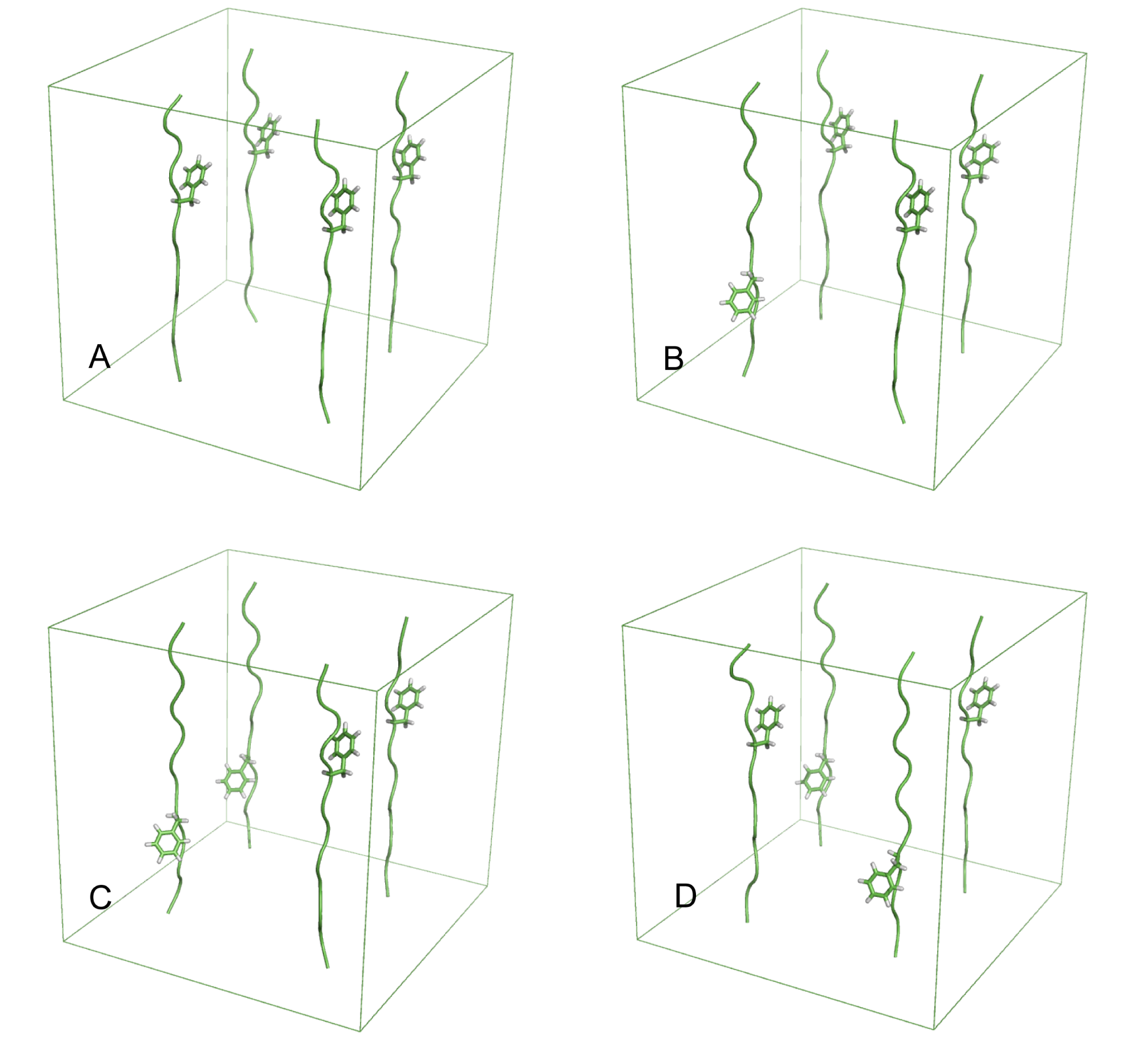

Supplement: Figure S1 — Initial arrangements of 4 identical hIAPP20–29 peptides. Backbones of peptides are shown in cartoon; side chains of Phe23 are explicitly represented in sticks for readily identification of N-terminals. (A) N-terminals of all four peptides point upwards; (B) N-terminals of three out of four peptides point upwards; (C) N-terminals of two peptides on one side point upwards; (D) N-terminals of two peptides on the diagonal point upwards. (0.94 MB TIF) [file pcbi.1000357.s001.tif]

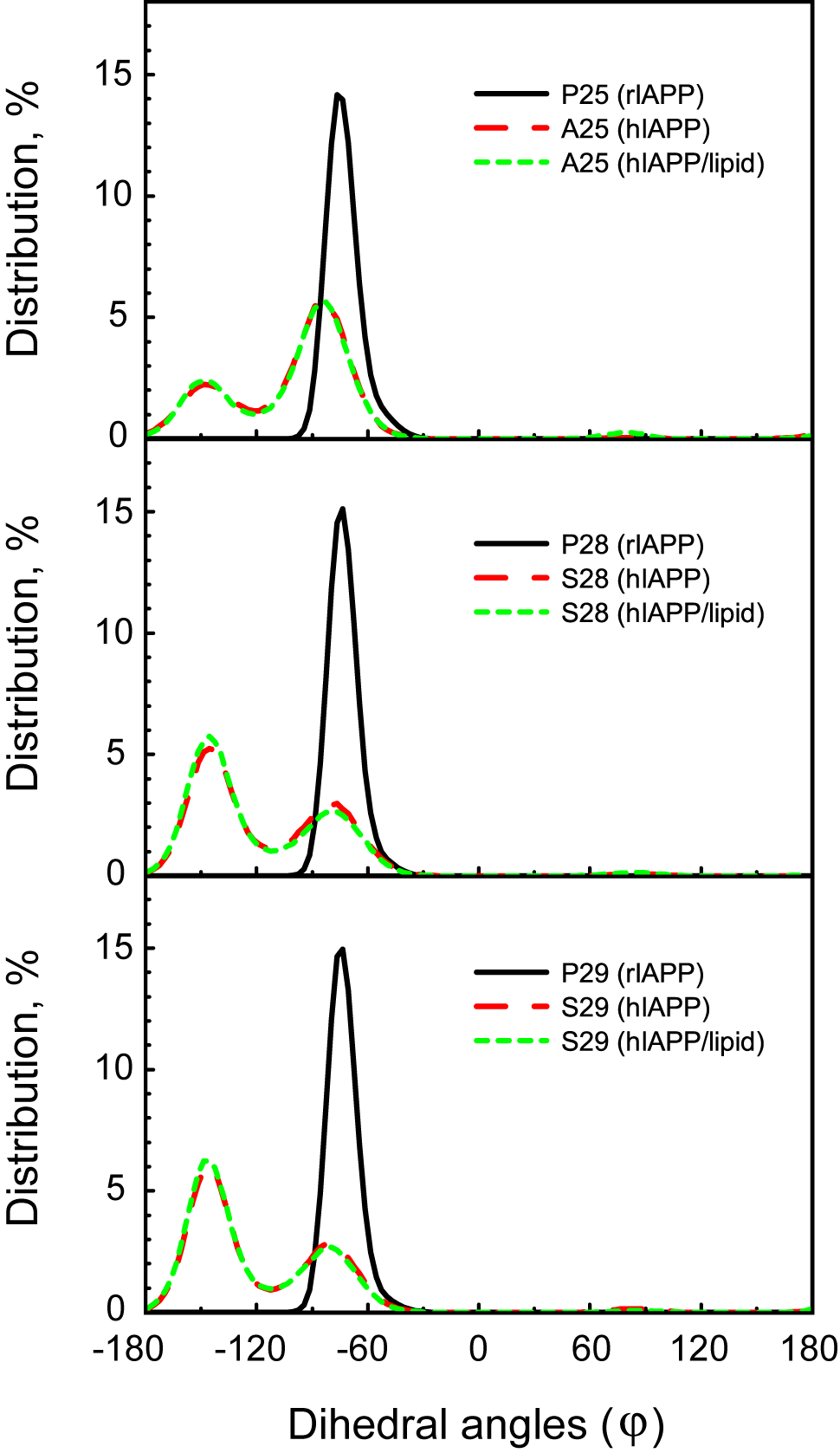

Supplement: Figure S2 — Distribution of ϕ dihedral angle of three prolines on rIAPP (black curves) and their counterpart residues on hIAPP (red curves) and hIAPP/lipid (green curves). (4.90 MB TIF) [file pcbi.1000357.s002.tif]
